# Gene amplification in the premalignant stages of non-small cell lung cancer development

**DOI:** 10.3389/fonc.2026.1803576

**Published:** 2026-05-20

**Authors:** Vanessa G. P. Souza, Katya H. Bénard, Greg L. Stewart, Katey S. S. Enfield, William W. Lockwood, Wan L. Lam

**Affiliations:** 1 British Columbia Cancer Research Institute, Vancouver, BC, Canada; 2 Interdisciplinary Oncology Program, University of British Columbia, Vancouver, BC, Canada; 3 Department of Pathology and Laboratory Medicine, University of British Columbia, Vancouver, BC, Canada

**Keywords:** cancer interception, early carcinogenesis, gene amplification, lung adenocarcinoma (LUAD), lung cancer, lung squamous cell carcinoma (LUSC), premalignant lesions, somatic copy-number alterations

## Abstract

Lung cancer remains the leading cause of cancer-related mortality worldwide. Over the past decade, major advances in targeted and immune-based therapies have improved outcomes for patients with advanced non-small cell lung cancer (NSCLC). However, further reducing mortality will require shifting intervention to the earliest, most curable stages of disease. Somatic copy-number alterations (SCNAs) are established drivers of NSCLC, and recent publications add to the growing evidence that gene amplifications, among the most frequent SCNAs in lung cancer, emerge early in carcinogenesis, including in premalignant and minimally invasive lesions. These early events can confer growth advantages that support clonal expansion, increase genomic instability, and propel progression toward invasive malignancy long before clinical diagnosis. Consequently, early gene amplifications represent a promising class of biomarkers for identifying high-risk lesions, refining risk stratification in screening settings, and exposing vulnerabilities amenable to early intervention. In this article, we synthesize current evidence on early amplification events in lung adenocarcinoma and lung squamous cell carcinoma, discuss their translational relevance for early detection and intervention, and outline key challenges and priorities for future research.

## 1 Introduction

Recent advances in targeted therapies and immune checkpoint inhibitors have improved outcomes in lung cancer; nevertheless, lung cancer remains the leading cause of cancer-related mortality worldwide (1). Major efforts have focused on detecting disease at the earliest clinical stages, when tumors are more likely to be curable and a broader range of therapeutic strategies can be applied (2).

Gene amplifications are among the most frequent genomic alterations in lung cancer and are well established as drivers of oncogenesis (3–10). Recent studies indicate that gene amplifications arise early during lung carcinogenesis, including in premalignant and minimally invasive lesions, and are essential for the transition to invasive growth (11–14). These findings coincide with emerging insights into translating premalignant cellular and molecular events associated with lung cancer for early detection and disease intervention (15, 16), thereby renewing interest in the earliest stages as clinically informative windows.

Therefore, early DNA amplification events represent a promising biomarker class with potential utility for risk stratification, early detection, and prevention-oriented intervention. In this article, we synthesize current evidence on early amplification events in lung adenocarcinoma (LUAD) and lung squamous cell carcinoma (LUSC), discuss their relevance for early detection and intervention, and outline key challenges and priorities for future research.

## 2 Gene amplification

### 2.1 Segmental DNA amplifications

Gene amplification is broadly defined as an increase in the number of copies of a gene (17). In cancer genomics, the term encompasses a spectrum of alterations with distinct biological and clinical significance. As used in this article, ‘gene amplification’ refers to somatic increases in the DNA copy-number of a genomic segment encompassing one or more genes. Within this category, we distinguish focal amplifications, defined as high-level, localized copy-number increases, from lower-level copy-number gains, including arm-level gains and whole-chromosome polysomy (18–20). Because thresholds for ‘amplification’ varies across studies, alteration type is reported wherever applicable.

Focal amplification of DNA increases the copy-number of genes within the amplified segment, which may result in dosage-dependent overexpression (5, 19, 21–29). Mechanisms of DNA amplification are well documented, such as breakage-fusion-bridge cycles and the formation of double minute chromosomes (24, 25, 30–35). More recently, a breakage-replication/fusion process has been proposed as a mechanism of segmental DNA amplification (36).

Oncogene amplification is a hallmark of aggressive tumor behavior, driving increased proliferation, invasion, and therapy resistance (11, 22, 34, 37–42). The capacity to undergo DNA amplification does not occur uniformly across tumors, and some cancers exhibit a heightened propensity for these events, known as the ‘amplifier phenotype,’ a propensity driven by underlying genomic instability (5, 43).

This instability is inherently imprecise; it generates large, complex amplicons that encompass driver oncogenes and neighboring segments of the genome. Consequently, while historical studies focused on the drivers, recent genomic landscape analyses have shown the significance of these co-amplified ‘passenger’ genes. Although not directly oncogenic, these passengers establish unique cellular stresses and dependencies, creating collateral vulnerabilities that represent actionable targets (23). Beyond protein-coding genes, amplifications can affect regulatory elements and non-coding RNA loci, further reshaping transcriptional programs and cellular states (44).

### 2.2 Lung cancer subtypes exhibit distinct amplicon profiles

**Figure 1:**
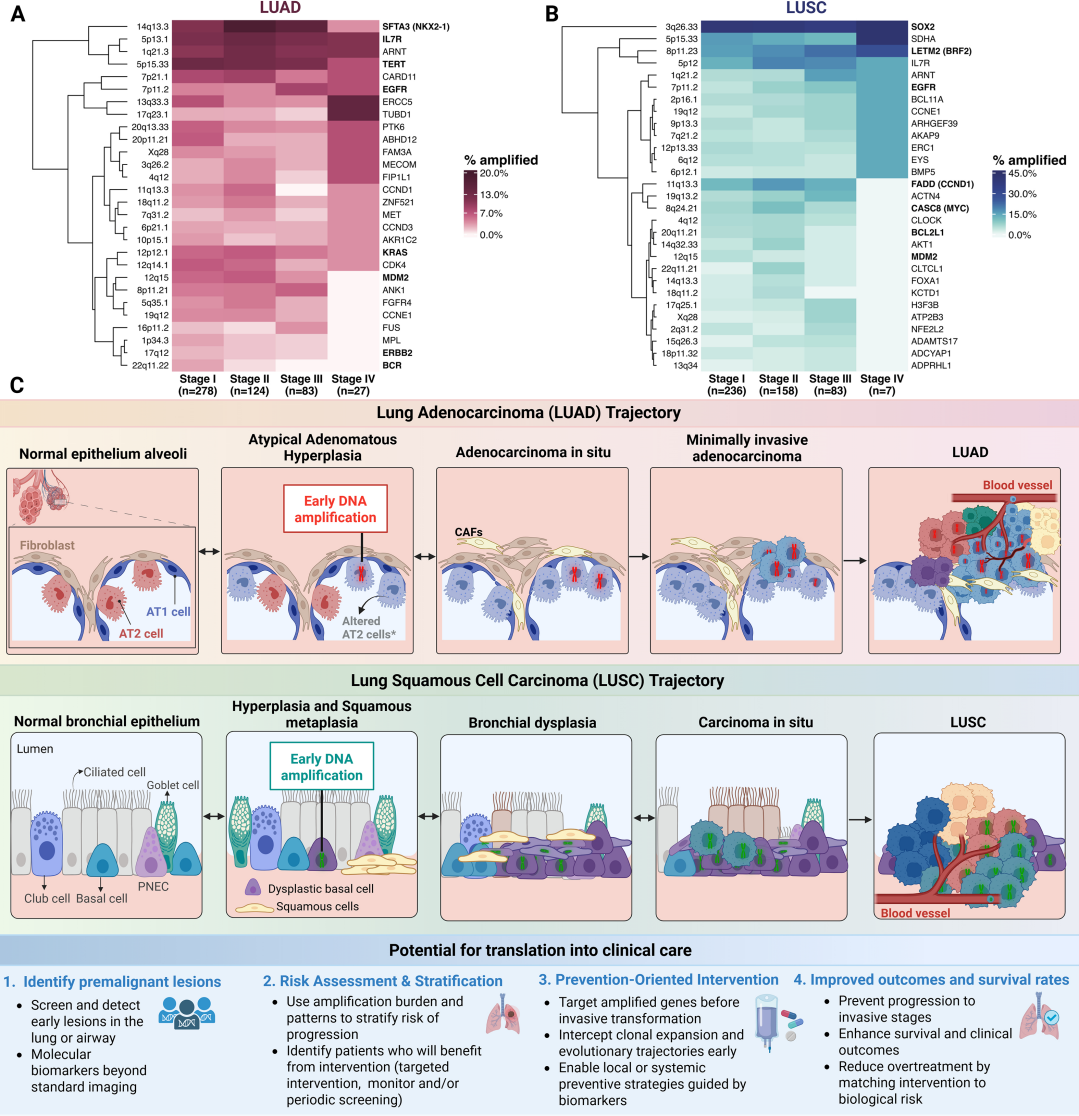
Stage-specific DNA copy-number amplifications and premalignant trajectories in lung adenocarcinoma (LUAD) and lung squamous cell carcinoma (LUSC). **(A, B)** Heatmaps showing the percentage of tumors with gene-level GISTIC2.0 high-level (+2) amplification calls, summarized at the level of GISTIC amplification peaks (cytobands) across pathological stages (I–IV) in the TCGA LUAD (left) and LUSC (right) cohorts. Each row represents a cytoband-level amplification peak defined in GISTIC peak outputs (confidence level 99%), clustered by similarity in stage-wise amplification frequency. Color intensity reflects the fraction of stage-specific tumors with ≥1 gene-level +2 amplification mapping to the indicated cytoband peak; the color scale is capped at the maximum observed frequency (rounded up to the nearest 5%) to preserve contrast. Representative genes are shown for annotation and were selected from within each peak’s gene list, prioritizing COSMIC Cancer Gene Census genes when present (126). Gene names discussed in the main text and/or listed in **Table 1** are shown in bold. Where the representative gene for a GISTIC peak differs from the gene discussed in the text, the latter is indicated in parentheses. Sample counts per stage are indicated below each column. Genomic coordinates, statistical significance, and full gene content of each amplicon are provided in **Supplementary Tables 1**–**S3** (LUAD) and **S4–S6** (LUSC). **(C)** Schematic representation of stepwise histological progression from normal epithelium to invasive carcinoma. In LUAD, normal alveolar epithelium progresses through atypical adenomatous hyperplasia (AAH), adenocarcinoma *in situ* (AIS) and minimally invasive adenocarcinoma (MIA) to invasive LUAD, with early gene amplifications highlighted in red in preinvasive and invasive lesions. *Altered AT2 cells refer to intermediate epithelial states arising during AT2 transdifferentiation (details can be found in (15) and (127)). AT1 cell: type I pneumocytes; AT2 cell: type II pneumocytes; and CAF: Cancer-associated fibroblasts. In LUSC, normal bronchial epithelium progresses through hyperplasia and squamous metaplasia, bronchial dysplasia, and carcinoma *in situ* (CIS) to invasive LUSC, amplification events in preinvasive stages are highlighted in green. Dysplastic basal cells refer to altered basal cells (details can be found in (15)). Arrows indicate the proposed stepwise progression between disease stages, with some early lesions having the potential to regress (denoted by bidirectional arrows). Bottom schematic: Potential for clinical translation of gene amplifications in premalignant lesions. Detection of these early genomic events enables the identification of high-risk lesions, allowing for risk stratification and the implementation of targeted, prevention-oriented interventions before malignant progression. Multi-panel figure comparing lung adenocarcinoma (LUAD) and lung squamous cell carcinoma (LUSC), showing stage-specific patterns of recurrent DNA copy-number amplification alongside schematic illustrations of premalignant-to-invasive disease progression and potential clinical applications of early genomic detection.

Recurrent amplification of distinct genes across different tumor types implies that specific genes confer advantages tied to cellular developmental context. This phenomenon, known as lineage-dependency, dictates that an amplicon provides a selective benefit when aligned with the tumor’s developmental context or cell-of-origin program (5, 21, 39, 43, 45). LUAD and LUSC, the two predominant histological subtypes of non-small cell lung cancer (NSCLC), are characterized by divergent cellular origins, risk factors, clinical outcomes, and distinct patterns of gene amplification (**Figure 1A**). LUAD frequently exhibits high-copy amplification of *EGFR*, or activating mutations, as well as focal amplification of *KRAS*, particularly the mutant allele (46–51). LUSC frequently exhibits high-level amplifications of *SOX2* and *TP63*, which function as lineage-survival oncogenes driving squamous differentiation and proliferation, and also often shows *MYC* amplification (52–56). Comprehensive genome-wide profiling showed that these subtype-specific amplicon landscapes are fundamental to the biological identity of tumors (57, 58).

## 3 Amplifications are early events in tumorigenesis

### 3.1 Premalignancy and early lung carcinogenesis

Lung cancer develops through a multistep evolutionary process where histologically defined premalignant lesions accumulate molecular alterations that ultimately enable invasive growth (15, 59–61).

Precursor lesions to LUAD arise in the distal lung parenchyma, where alveoli are lined by type I (AT1) and type II (AT2) pneumocytes (15, 62). AT2 cells have stem-like properties, regenerating alveolar epithelium after injury and producing pulmonary surfactant (62). Premalignant lesions in LUAD include atypical adenomatous hyperplasia (AAH) and adenocarcinoma *in situ* (AIS) (63). AAH is characterized by small proliferative lesions composed of mainly atypical AT2 cells that grow along intact alveolar septa in a lepidic pattern. AIS shares these features and is regarded as continued growth from AAH, but is distinguished from AAH by size (63). AIS is further distinguished from minimally invasive adenocarcinoma (MIA), which is defined by limited invasive components within an otherwise lepidic lesion (15, 63).

LUSC development occurs in the conducting airways from bronchial epithelium primarily composed of basal, secretory, and ciliated cells (15, 64). Basal cells serve as key airway stem cells during homeostasis and repair and are regarded as the predominant cell of origin for LUSC. Tobacco-related injury can promote cell-state changes and drive expansion of basal or basal-like populations. Premalignant progression includes hyperplasia and squamous metaplasia, followed by graded squamous dysplasia and carcinoma *in situ* (CIS). While early lesions may regress, persistent high-grade dysplasia and CIS are associated with increased risk of progression (15, 64–66).

### 3.2 Premalignant molecular alterations: a window of opportunity

Early detection and timely intervention represent critical factors for patient survival in lung cancer (67). The importance of expanding the understanding of the molecular mechanisms driving these lesions is key to developing innovative modalities to detect, stratify, and intercept lung cancer at its nascent stages (16). Recent studies have elucidated the genomic, epigenomic, and transcriptomic landscapes of preinvasive lung cancer lesions (68–71). These works add to the evidence that preinvasive lesions harbor molecular alterations including mutations, somatic copy-number alterations (SCNAs), altered gene expression, methylation, and others (70–76). Translating this knowledge into tools for the detection, prediction, and early intervention remains an unmet need (15, 16).

### 3.3 Early arising gene amplifications in preinvasive lung lesions

Although SCNAs have been catalogued in stage I–IV LUAD and LUSC tumors (**Figure 1**) (77–81), their contribution to premalignant evolution has historically received less attention even though they may drive clonal expansion and diversification (68, 82–85). One proposed mechanism is ‘self-promotion,’ whereby evolving copy-number gains endow initiating cells with promoter-like properties by (i) increasing the dosage of genes that sustain proliferative signalling, (ii) preferentially amplifying mutant alleles of early driver variants, and (iii) accelerating acquisition of additional genomic alterations (83).

Consistent with this framework, mathematical models and empirical cancer genome studies support a punctuated mode of genomic evolution, with early bursts of SCNAs followed by relative clonal stability and expansion (86–89). Multi-omics profiling of preinvasive lung lesions confirms that SCNAs (including focal amplifications) are already present and may shape the early carcinogenic trajectories (68, 71, 90).

### 3.4 Early amplifications in lung adenocarcinoma

In recent studies of preinvasive and minimally invasive LUAD precursor lesions (including AAH/AIS/MIA) 14q13.3 amplification, encompassing the LUAD lineage-specific *NKX2*-1 (*TTF-1*) gene, a master transcriptional regulator of lung morphogenesis, emerges as a defining early event (12, 68–71). Multi-region sequencing from the TRACERx cohort confirms that *NKX2–1* focal copy-number gains often arise early in tumor evolution (91).

Beyond *NKX2-1*, the preinvasive landscape of LUAD is characterized by a specific repertoire of discrete focal amplifications – for example at 12q15 (*MDM2*), 8q24 (*MYC*), 5p15 (*TERT*), 12p12 (*KRAS*), and 7q21 (*CDK6*) – that arise before the onset of broad chromosomal instability (**Table 1**) (12, 69–71). While most of these loci also exhibit amplification peaks in invasive tumors, *CDK6* shows distinctive patterns, with significant amplification peaks detected in preinvasive lesions (71).

**Table 1:** Gene amplifications and somatic copy-number gains in premalignant lung lesions.

Lung adenocarcinoma (LUAD)			
Locus amplified	Study description	Lesion(s)	Reference
5p, 7p, 8p, 8q, 16p, 17q (arm)	Recurrent arm-level CN gains observed across preinvasive lesions, consistent with early aneuploidy.	AAH/AIS/MIA	(12)
*IL7R* (5p13.2)	Recurrent CN gains identified by multiregional exome sequencing.	MIA	(70)
*PDCD6* (5p15.2)	qPCR-validated *PDCD6* gain; increased CN in lesions with invasive components vs non-invasive BAC	AIS	(95)
*TERT* (5p15.33)	Focal amplification in AIS/MIA; chr 5p polysomy; increased *TERT* CN in lesions with invasive components.	AIS/MIA	(71, 95)
*NOTCH4* (6p21.32)	CN amplification detected in MIA, enriched in the invasive focus by multiregional targeted sequencing.	MIA	(76)
*EGFR* (7p11.2)	Low-level copy-number gain (polysomy; >3 *EGFR* signals per nucleus) observed in early lesions, with higher-level *EGFR* amplification detected more frequently in invasive adenocarcinoma, consistent with CN increase as a progression-associated event.	AAH/AIS/MIA	(70, 94, 96)
*KRAS* (12p12.1)	Gain of chromosome 12p12.1 encompassing *KRAS* is detected in AIS/MIA and recurrently observed across AIS/MIA/ADC.	AIS/MIA	(70, 71)
*RIT1* (1q22)	Focal gain identified by multiregional exome sequencing.	AIS	(70)
*PIK3CA* (3q26.3)	Copy-number gain involving the PI3K pathway component *PIK3CA* detected in early adenocarcinoma development.	AIS/MIA	(70)
*CDK6* (7q21.2)	Focal amplification of the cell-cycle regulator CDK6 detected in preinvasive lesions.	AIS/MIA	(71)
*SMO* (7q32.1)	CN gain at the *SMO* locus observed in a subset of early lesions.	MIA	(70)
*BRAF* (7q34)	CN gain involving the MAPK pathway component BRAF detected in early-stage lesions.	AAH/MIA	(70)
*MYC* (8q24.21)	Focal amplification detected in early lesions, with higher frequency observed in invasive LUAD.	AIS/MIA	(71)
*MDM2* (12q15)	Focal amplification detected by WES and RNA-seq; more frequent in invasive LUAD.	AIS/MIA	(71)
*NKX2-1* (14q13.3)	Focal amplification at the chr14q13.3/*NKX2–*1 locus detected in AIS/MIA; recurrent LUAD amplicon consistent with lineage-associated oncogenic dependency.	AIS/MIA	(3, 71)
*PML* (15q24.1)	CN gain involving *PML* detected in early lesions.	MIA	(70)
*BTK* (Xq21.33)	CN gain detected in preinvasive lesions by multiregional exome sequencing.	AIS/MIA	(70)
Lung squamous cell carcinoma (LUSC)			
*Locus amplified*	*Study description*	*Lesion(s)*	*Reference*
3q, 5p, 8q, 19q (arm)	Whole-genome sequencing of bronchial CIS reveals widespread aneuploidy mirroring invasive LUSC; copy-number alterations are significantly more frequent in progressive versus regressive lesions.	CIS	(68)
*TP63* (3q28), *MYC* (8q24), *EGFR* (7p12), 5p15.2	FISH-detected recurrent low-level CN gains (mean >2 copies/cell) reflecting chromosomal aneusomy; ≥3 abnormal markers strongly associated with lung cancer risk (adjusted OR ≈17).	Moderate–Severe Dysplasia/CIS	(14)
*WNT4* (1p36.12)	0.4 Mb amplified region identified in preinvasive squamous samples.	Dysplasia/CIS	(97)
*RASGRP3* (2p22.3)	Recurrent 2p22.3 amplification (3.1 Mb, 13 genes) detected by SNP-array analysis in 3/6 preinvasive lesions; qPCR-validated for *RASGRP3*; novel early CN alteration in squamous carcinogenesis.	Moderate–Severe dysplasia/CIS	(98)
*GDNF* (5p13.2)	Focal ~0.34 Mb amplification in bronchial CIS; transcript and protein overexpression in CIS and invasive tumors.	CIS	(99)
*TRIO* (5p15.2)	Focal ~0.27 Mb amplification detected in bronchial CIS and maintained in invasive squamous carcinoma; CN gain correlates with *TRIO* overexpression.	CIS	(99)
*BRF2* (8p12)	Focal amplification detected in ~35% of bronchial CIS; validated early squamous oncogene.	Dysplasia/CIS	(100)
3q26.2-q29	3q26 gain (amplification or polysomy) is confined to high-grade squamous lesions and absent in low-grade lesions; longitudinal profiling shows 3q26.2–q29 gain occurs at the site of future cancer, persists during progression from squamous metaplasia to CIS/SCC, and predicts endobronchial cancer risk.	Squamous metaplasia/Severe dysplasia/CIS	(101, 102)
*PIK3CA* (3q26.32)	Amplified in all high-grade lesions tested; within 3q amplicon; co-amplified with *SOX2*.	Severe dysplasia/CIS	(11)
*SOX2* (3q26.33)	3q copy-number gain increases with squamous lesion grade; low-level *SOX2* gain can be detected in dysplasia, while focal *SOX2* amplification is restricted to a subset of high-grade dysplasia/CIS and is enriched in lesions clonally linked to invasive SCC, consistent with a progression-associated event.	Severe dysplasia/CIS	(11, 103, 104)
*TP63* (3q27–3q28)	FISH-detected genomic amplification in severe dysplasia/CIS, rare in low-grade lesions; associated with ΔNp63α overexpression and retained in invasive SCC, where it correlates with favorable survival.	Severe dysplasia/CIS	(105)

A systematic literature search was performed in PubMed up to January 23, 2026, to identify studies reporting somatic copy-number (CN) alterations, with a particular focus on focal amplifications, in preinvasive lung lesions. Search terms were organized into three concept blocks: (i) copy-number and amplification–related terms (e.g., amplification, copy-number variation, somatic copy-number alterations); (ii) histologically defined premalignant and preinvasive lung lesions; and (iii) lung-specific context. To account for the distinct evolutionary pathways of lung adenocarcinoma (LUAD) and lung squamous cell carcinoma (LUSC), separate searches were conducted for each histologic subtype. For LUAD, lesion-specific terms included atypical adenomatous hyperplasia (AAH), adenocarcinoma *in situ* (AIS), and minimally invasive adenocarcinoma (MIA), along with broader descriptors such as preinvasive and premalignant. For LUSC, searches incorporated bronchial dysplasia and carcinoma *in situ* (CIS). All lesion-related terms were combined with lung-specific keywords and amplification-related terms using the AND operator, while synonymous terms within each block were combined using OR. Searches were restricted to titles and abstracts to enhance specificity. Retrieved records were screened manually; studies were included if they reported copy-number gains or gene amplifications detected in histologically defined preinvasive human tissue.

Findings from historical studies using the term bronchioloalveolar carcinoma (BAC) were harmonized with contemporary terminology. Lesions described as pure BAC were treated as AIS-equivalent, whereas BAC-associated mixed or minimally invasive lesions were mapped to AIS or MIA where supported by the original histologic descriptions, in accordance with the 2015/2021 WHO Classification (106, 107). This reclassification was applied conservatively to preserve biological intent while ensuring clinical relevance.

As defined in Section 2.1, ‘amplification’ criteria vary by study; the study description specifies whether the reported event represents focal high-level amplification, low-level gain, aneusomy, or polysomy, with broad arm-level gains listed as general chromosomal arms (e.g., 5p) and focal alterations listed with specific cytobands (e.g., 14q13.3). Where a single locus encompasses more than one type of alteration (e.g., 3q26 gain), this is noted accordingly. Rows are ordered by chromosomal location, beginning with arm-level and multi-locus entries, followed by all p arm loci then all q arm loci, each in chromosome order from centromere to telomere (low to high band numbers).

The frequency and magnitude of these focal amplifications display a stepwise increase corresponding to histological progression. While focal gains of *TERT*, *MDM2*, and *KRAS* are identified in a subset of AIS and MIA lesions, their prevalence significantly expands in invasive adenocarcinoma (71). Similarly, focal amplification of the 8q24 locus (*MYC*) is detectable in preinvasive specimens but becomes a dominant feature in established tumors (71). *MYC* and *MDM2* amplifications are prognostic markers for early stage LUAD (92, 93).

While *EGFR* and *KRAS* rank among the most frequently mutated genes in LUAD (51), high-level focal amplification of these genes is not common in preinvasive lesions (71). Chromogenic *in situ* hybridization identified increased *EGFR* copy-number in a subset of AAH lesions, whereas higher copy-number states were more frequent in invasive adenocarcinoma (94).

### 3.5 Early amplifications in lung squamous cell carcinoma

The gain of distal 3q represents the most frequent SCNA in LUSC and constitutes one of the most striking genomic distinctions between LUSC and LUAD, in which this event is rare (57, 108–110). 3q gain in LUSC is frequently focal, converging on a minimal common region at 3q26–3q28 containing approximately 25 co-amplified genes, establishing this locus as a hallmark early driver of squamous carcinogenesis (110).

The 3q amplicon encompasses key lineage-defining oncogenes, most notably *SOX2*, *TP63*, and *PIK3CA*, which collectively enforce the squamous transcriptional and signaling program. Multiple studies have shown that 3q amplification is an early, initiating event detectable in preinvasive squamous lesions, including CIS and high-grade bronchial dysplasia (11, 101, 103). Longitudinal analyses of bronchial premalignant lesions demonstrate that 3q gain serves as a robust biomarker of malignant potential: while largely absent from low-grade lesions, it is detected in most high-grade dysplasias and CIS, with its presence predicting progression to invasive carcinoma (11). *SOX2* overexpression initiates preinvasive-like squamous phenotypes in human bronchial epithelial cell models and cooperates with *PI3K* and oxidative stress pathway dysregulation to promote invasive properties (111).

Overexpression of *CCND1* at 11q13 represents another recurrent alteration in LUSC development. *CCND1* is a rate-limiting regulator of G1–S cell-cycle progression (112, 113), and its overexpression is due to 11q13 amplification in some cases (114–118). Histopathological studies demonstrate a stepwise increase in *CCND1* expression with increasing lesion severity, from metaplasia through dysplasia to invasive carcinoma (119, 120). Elevated *CCND1* expression is also detected in histologically normal epithelium adjacent to tumors, consistent with a role in early field cancerization (121–123).

Additionally, focal amplifications on 8p12 have been identified as an early event in LUSC evolution. The 8p12 amplicon is present in many invasive LUSC tumors and is largely absent or rare in LUAD (57, 124, 125). Integrative analyses have identified *BRF2* as a lineage-specific oncogene within this region (100). *BRF2* undergoes focal amplification and overexpression in >35% of preinvasive bronchial carcinoma *in situ* and in dysplastic lesions (100). A list of amplified genes described in premalignant lesions of LUSC is provided in **Table 1**.

### 3.6 Focal amplification precedes invasion across multiple solid tumor types

The phenomenon of focal amplification in precancer stages is not unique to lung carcinogenesis; evidence from other solid tumor models confirms that these genomic events often precede malignant invasion (128–132). In breast cancer, *ERBB2* (HER2) amplification is identified in ~40–50% of ductal carcinoma *in situ* (DCIS) lesions and persists in ~15–20% of invasive carcinomas (133–137). In esophageal adenocarcinoma, amplifications of *CCND1* and *ERBB2* are distinct markers of progression in Barrett’s esophagus, emerging in high-grade dysplasia (128, 138). These focal amplifications distinguish aggressive clones, with *ERBB2* and *CCND1* overexpression linked to cell cycle dysregulation and evasion of apoptosis during the metaplasia-dysplasia-carcinoma sequence (128). In head and neck squamous cell carcinoma (HNSCC), *EGFR* amplification is a recurrent event in oral leukoplakia, where its presence predicts transformation to invasive carcinoma (139–141).

### 3.7 The transient nature of focal amplifications

Unlike point mutations, which tend to be stable and heritable (91, 142), early focal amplifications can display a ‘disappearing’ phenotype, being present in precursor lesions but absent or less frequent in invasive tumors. In NSCLC, this is exemplified by the lineage-survival oncogene *NKX2-1*. While frequently amplified in early-stage LUAD to enforce lineage survival, *NKX2–1* can act as a metastasis suppressor in later stages; consequently, invasive subclones may downregulate its expression or lose the focal amplification to facilitate dedifferentiation and dissemination (143–145). **Figure 1** illustrates stage-wise differences in the prevalence of focal amplifications in LUAD and LUSC tumors across stages I-IV. While drivers like 3q26.33 in LUSC and 5p13.1 (*IL7R*) in LUAD show comparatively persistent high frequency across the stages, other loci, such as 22q11.22 (*BCR*) in LUAD and 20q11.21 (*BCL2L1*) in LUSC, exhibit a visible reduction in frequency as the disease progresses to Stage IV.

Mechanistically, this plasticity may be partially attributed to the presence of amplicons on extrachromosomal DNA (ecDNA) (37). Lacking centromeres, ecDNA segregates unevenly during mitosis, allowing tumor cells to rapidly accumulate high copy numbers to fuel early growth (37). Classical studies demonstrate that double minute–based amplifications can be unstable without selective pressure (e.g., loss of amplified drug-resistance loci after withdrawal of the selecting agent) (146, 147). Moreover, double minutes can be actively eliminated through micronucleation, nuclear budding, or selective depletion following replication stress/DNA damage (32, 148), providing plausible routes for ‘reversible’ amplification states.

This creates a ‘genomic blind spot’ since they can be missed (given that most studies focus on invasive stages where the initiating subclone may have been replaced by a clone driven by different alterations). By the time NSCLC becomes clinically invasive, the genome has typically acquired a high tumor mutational burden and subclonal diversity (70), sometimes ‘swallowing’ or obscuring the initial tumor driver events (69, 91, 149).

## 4 Translational potential and future directions

While low-dose CT (LDCT) screening has reduced NSCLC mortality by identifying indeterminate pulmonary nodules and lesions at earlier stages, it also highlights the need to determine which lesions will progress and which patients are most at risk (150–157). Large-scale screening studies are required to identify novel, clinically relevant amplifications in early lesions. Incorporating longitudinal patient follow-up will enable association of these alterations with NSCLC risk and disease progression.

Alternative methods could make amplification detection more clinically practical. While the current clinical standard of amplification detection, fluorescence *in situ* hybridization (FISH), is low throughput, recent work has shown that using NGS effectively distinguishes *ERBB2* amplified from non-amplified cases of NSCLC (158). Another strategy to enhance clinical applicability is the incorporation of novel gene amplifications into liquid biopsy–based approaches. Recent evidence demonstrates that amplification of *MET* and *ERBB2* can be reliably detected in circulating tumor DNA (ctDNA) from patients with NSCLC, highlighting the potential of copy-number alterations as clinically actionable biomarkers (159). Multi-omics approaches may enhance detection of relevant features by allowing the simultaneous interrogation of DNA alterations and DNA methylation changes within a single platform, as demonstrated by recent cell-free DNA studies (160, 161). In fact, DNA methylation patterns are being investigated as clinically informative biomarkers in ctDNA studies across numerous cancers (162, 163).

While research on clinical interception strategies is a novel and ongoing area, there are several areas where detection of gene amplifications in early lung lesions may provide actionable insights. Amplification status could be used to identify high-risk individuals who would benefit from more regular screening, and patients with clinically actionable amplifications may be ideal candidates for early intervention using existing targeted therapies directed against amplified oncogenes (164, 165). Additionally, amplification patterns could be used to predict resistance or sensitivities to targeted therapeutics (166). Since gene amplification is only one of several mechanisms of oncogene activation, in addition to focusing on individual oncogenes, the presence of focal segmental DNA amplification may also be considered a phenomenon or a ‘mutator phenotype’ (167) that could serve as a marker for elevated genomic instability and increased cancer risk (168, 169).

Lastly, relevant amplifications could be harnessed for patient enrollment into biomarker-enriched clinical trials designed to evaluate interception strategies, supported by evidence that biomarker integration improves clinical trial efficiency and likelihood of success (170).

While copy-number losses fall outside the scope of this study, it is worth acknowledging that they represent an important dimension of the SCNA landscape of premalignant stages of NSCLC (70, 171–174). Among these, 3p21.3 deletions are one of the earliest and most common genetic events in lung cancer development, particularly in LUSC (171, 174). In invasive NSCLC, well-known losses include deletions at the 9p21.3 locus linked to poorer prognosis (175), immune evasion, metastasis (176), and recurrence in early-stage LUAD (177). The translational relevance of deletions for early detection and intervention, as well as their potential as priorities for future research, are worth exploring in dedicated studies.

Future studies of preinvasive and minimally invasive stages of lung cancer should also account for etiological context. While the composition of SCNAs differs between smoking-related and never-smoker invasive NSCLC (177–181), there is evidence that in preinvasive stages SCNAs may be found in the bronchial epithelium of smokers (with and without cancer) but not in never-smokers (171), indicating an early smoking-associated field defect preceding invasive disease. These differences may inform etiology-specific early detection and risk stratification strategies and deserve further attention.

In addition, the role of ecDNA in early amplification events warrants further exploration. ecDNA can underlie the plasticity of early focal amplifications, and recent evidence from esophageal adenocarcinoma demonstrates that ecDNA can arise during the precancer-to-cancer transition and is associated with disease progression (182). Given that ecDNA drives intra-tumoral copy-number heterogeneity and is associated with poor patient outcomes, ecDNA status may represent an additional metric for risk stratification in early lung lesions (183, 184). The unique properties of ecDNA, including lack of centromeres, open chromatin structure, and pervasive replication stress, create selective vulnerabilities that are being targeted therapeutically, with the first ecDNA-directed therapy currently in phase I clinical trials across multiple cancer types including lung cancer (183–186). Determining how ecDNA contributes to preinvasive lung carcinogenesis remains an important priority.

Finally, beyond DNA-based liquid biopsy approaches, expanding detection strategies to include metabolic biomarkers represents a promising complementary direction. Tumor-derived volatile organic compounds detectable in exhaled breath have shown high diagnostic performance for stage I lung cancer detection. Growing evidence suggests that genomic alterations, including structural variants and their accompanying passenger alterations, may drive distinct oncometabolic profiles with diagnostic potential (187–190), representing an emerging, translationally relevant avenue warranting investigation as a cost-effective, minimally invasive strategy for early detection and risk stratification in preinvasive lung cancer.

## 5 Conclusion

Early gene amplifications represent a fundamental yet underappreciated aspect of lung cancer evolution. Our review demonstrates that these events frequently arise in premalignant lesions, often preceding invasive transformation. Their transient and subclonal nature means many are lost, masked, or under-recognized as tumors evolve, particularly because most studies focus on established tumors. With the advent of high-throughput sequencing and single-cell genomics, it is now possible to systematically interrogate early amplifications with unprecedented resolution. Defining and understanding these early drivers could reveal a new class of biomarkers for early detection, risk stratification, and cancer intervention.

## Glossary

AAH: Atypical adenomatous hyperplasia
AIS: Adenocarcinoma in situ
Amplicon: A segment of DNA that is amplified as a result of genomic amplification
AT1: Alveolar type I cell
AT2: Alveolar type II cell
BCL2L1: BCL2 like 1
BRAF: B-Raf proto-oncogene, serine/threonine kinase
BRF2: BRF2 general transcription factor IIIB subunit
BTK: Bruton tyrosine kinase
CCND1: Cyclin D1
CDK6: Cyclin-dependent kinase 6
CIS: Carcinoma in situ
CN: Copy-number
CAN: Copy-number alteration
COSMIC: Catalogue Of Somatic Mutations In Cancer
ctDNA: Circulating tumor DNA
ecDNA: Extrachromosomal DNA
EGFR: Epidermal growth factor receptor
ERBB2: Erb-B2 receptor tyrosine kinase 2
FISH: Fluorescence *in situ* hybridization
GDNF: Glial cell line–derived neurotrophic factor
GISTIC: Genomic Identification of Significant Targets in Cancer
HNSCC: Head and neck squamous cell carcinoma
IL7R: Interleukin 7 receptor
KRAS: KRAS proto-oncogene, GTPase
LDCT: Low-dose computed tomography
LUAD: Lung adenocarcinoma
LUSC: Lung squamous cell carcinoma
MDM2: MDM2 proto-oncogene, E3 ubiquitin protein ligase
MIA: Minimally invasive adenocarcinoma
MYC: MYC proto-oncogene, bHLH transcription factor
NGS: Next-generation sequencing
NKX2-1: NK2 homeobox 1
NOTCH4: Notch receptor 4
NSCLC: Non-small cell lung cancer
PDCD6: Programmed cell death 6
PIK3CA: Phosphatidylinositol-4,5-bisphosphate 3-kinase catalytic subunit alpha
PML: PML nuclear body scaffold
RIT1: Ras like without CAAX 1
SCNA: Somatic copy-number alteration
SqM: Squamous metaplasia
SMO: Smoothened, frizzled class receptor
SOX2: SRY-box transcription factor 2
TERT: Telomerase reverse transcriptase
TP63: Tumor protein p63
TRACERx: Tracking Cancer Evolution through Therapy
TRIO: Trio Rho guanine nucleotide exchange factor
WES: Whole-exome sequencing
WGS: Whole-genome sequencing
WNT4: Wnt family member 4.
